# Sustained and transient attentional processes modulate neural predictors of memory encoding in consecutive time periods

**DOI:** 10.1002/brb3.150

**Published:** 2013-06-16

**Authors:** Tullia Padovani, Thomas Koenig, Doris Eckstein, Walter J Perrig

**Affiliations:** 1 Institute of Psychology, University of BernBern, Switzerland; 2 Center for Cognition, Learning, and Memory (CCLM), University of BernBern, Switzerland; 3 University Hospital of Psychiatry, University of BernBern, Switzerland

**Keywords:** Memory encoding, neural predictors, SME, sustained, task switching, transient attentional mechanisms

## Abstract

Memory formation is commonly thought to rely on brain activity following an event. Yet, recent research has shown that even brain activity previous to an event can predict later recollection (subsequent memory effect, SME). In order to investigate the attentional sources of the SME, event-related potentials (ERPs) elicited by task cues preceding target words were recorded in a switched task paradigm that was followed by a surprise recognition test. Stay trials, that is, those with the same task as the previous trial, were contrasted with switch trials, which included a task switch compared to the previous trial. The underlying assumption was that sustained attention would be dominant in stay trials and that transient attentional reconfiguration processes would be dominant in switch trials. To determine the SME, local and global statistics of scalp electric fields were used to identify differences between subsequently remembered and forgotten items. Results showed that the SME in stay trials occurred in a time window from 2 to 1 sec before target onset, whereas the SME in switch trials occurred subsequently, in a time window from 1 to 0 sec before target onset. Both SMEs showed a frontal negativity resembling the topography of previously reported effects, which suggests that sustained and transient attentional processes contribute to the prestimulus SME in consecutive time periods.

## Introduction

Attention and memory are fundamental cognitive processes of human intellectual function. Despite their interdependence, they are mostly investigated as separate processes. Recent neuroimaging studies have shown a tight relation between attentional control mechanisms and episodic memory (Chun and Turk-Browne 2007; Cabeza et al. 2008; Uncapher and Rugg 2009), demonstrating the role of attentional selection and modulation on memory encoding. It is well known that focusing and attending to a stimulus or an event greatly increases the probability to encode and retain this information (Yi et al. 2004; Kandel 2006). Attending implies both selecting the relevant information and allocating the processing resources to perform the related task (Craik et al. 1996; Chun and Turk-Browne 2007). Several researchers have started to examine these attentional mechanisms by investigating whether pretrial activity predicts retrieval success.

Even if memory encoding is generally thought to rely on brain activity following an event, an increasing line of evidence shows that prestimulus event-related potential (ERP) activity predicts later recollection (SME, subsequent memory effect), highlighting the role of the activity preceding stimulus presentation in the formation of a lasting memory (Otten et al. 2006, 2010; Gruber and Otten 2010; Padovani et al. 2011). In order to investigate preparatory activity, all these studies focused on the neural activity in the time window between the presentation of different cue types and the stimulus onset. The cues switched randomly across trials and indicated which type of task to perform on the upcoming stimulus. The resulting pattern of this preparatory encoding-related activation is characterized by a more pronounced frontal ERP negativity for later remembered versus later forgotten trials (Otten et al. 2006, 2010; Padovani et al. 2011).

The debate about the mechanisms through which prestimulus activity modulates memory encoding is still ongoing. Therefore, the aim of the current study was to clarify if both sustained and transient attentional processes modulate the prestimulus SME and if so, to determine the timing of their influence. On a theoretical level, these two types of attentional processes are generally described as executive attentional networks as for instance in the dual network model of attentional control (Dosenbach et al. 2008; Petersen and Posner 2012) and reflect different aspects of cognitive control. Sustained attentional processes prevail during repeated task sequences and reflect active maintenance demands associated with keeping multiple task sets available and/or engaging attentional monitoring processes to enhance sensitivity to environmental changes, induced for instance by cues (Braver et al. 2003). On the other hand, task switching sequences reflect mainly transient control processes associated with the change of the tasks, such as the reconfiguration and/or the updating of goals, leading to an appropriate reaction for the current task (Meiran et al. 2000; Braver et al. 2003; Monsell 2003; Reynolds et al. 2004).

In this paper, we focus on the prestimulus brain activity and on its role in memory formation. In order to investigate different aspects of this activity with the subsequent memory paradigm, we have performed two different studies using the same data set. In the first study, we aimed to identify different types of SMEs for emotional and semantic tasks (Padovani et al. 2011). In the current study we focus on the circumstances favoring the emergence of the prestimulus SME and investigate the relation between transient and sustained attentional processes and successful encoding. Two conditions were compared, which differed with respect to whether the task of the preceding trial was the same (stay condition) or different (switch condition). This method allowed us to determine in which of the two conditions the typical frontal negativity pattern of the prestimulus SME would be observed. This frontal negativity is thought to reflect the involvement of the left inferior prefrontal cortex (LIPC) in episodic encoding and is associated with subsequent memory recognition (Wagner et al. 1998, 1999; Otten et al. 2001).

Involvement of the prefrontal cortex (PFC) in prestimulus effects has been reported in a series of functional magnetic resonance imaging (fMRI) studies showing the crucial role of prefrontal cortex in processing of future events (Sakai and Passingham 2003, 2006; Haynes et al. 2007). In a first study using a task cueing paradigm similar to the one presented in this article, Sakai and colleagues identified the neural correlates of task sets, showing a pretask activation in the PFC related to the preparation of a specific task. The authors interpreted this finding associating the activation of the anterior part of the PFC to the construction of higher order representations that are involved in the preparation of future task operations even without specific task items (Sakai and Passingham 2003). In a following study they showed the existence of a mechanism in the PFC that is involved in the representation of task rules and revealed how this mechanism modulates subsequent cognitive performance through a rule-specific neural activity before the task execution (Sakai and Passingham 2006). In a third study, they demonstrated the possibility to infer from the activity of medial and lateral regions of the PFC which of two tasks the subjects were intending to perform, showing that this area encodes intention-related information specific to the preparation of the future task (Haynes et al. 2007). Besides the involvement of PFC, midbrain and medial temporal regions were shown to play a role in predicting later recollection (Mackiewicz et al. 2006; Park and Rugg 2010).

In a direct comparison of the influence of transient and sustained attentional processes on successful encoding, Reynolds et al. (2004) investigated the relation between item and task level processes and reported evidence for an enhanced activation of the PFC during transient attention. Their results showed greater activation in the LIPC during task switching (task change at every trial) compared to the single task condition (same task throughout a block) and for subsequently remembered versus forgotten items. Further findings showing an increased activation of PFC during transient versus sustained attentional processes can be also found in the task switching research literature (Braver et al. 2003; Gladwin et al. 2006).

These findings are in line with the hypothesis that shared PFC recruitment during episodic encoding might reflect a functional overlap of working memory (WM) and cognitive control processes (Wagner 1999). This hypothesis was actually tested with an fMRI study evaluating the overlap between sustained and transient activation patterns across long-term memory (LTM), attentional, and WM tasks. The results confirmed the shared PFC recruitments in WM and LTM tasks only for transient item-related responses (Marklund et al. 2007). Therefore, there is substantial evidence that the SME is related to transient attentional processes.

Furthermore, there is also some empirical proof for an involvement of prefrontal activation and frontal negativity in sustained attention, even in task switching paradigms, where it is important to maintain internal representations of multiple task sets over a prolonged time (Braver et al. 2003; Barcelo et al. 2006; Gladwin et al. 2006).

The few studies, however, that directly compared the ERPs of task switching with task repetition provided mixed results (Wylie et al. 2003; Gladwin et al. 2006). It is therefore still unclear, as to what extent sustained attention contributes to the SME. Another study on encoding-related activity using a task switch paradigm showed the contribution of both sustained and transient attentional processes in the determination of the SME (Otten et al. 2010). However, the findings did not reveal any difference between the effects of stay and switch trials, associated, respectively, with sustained and transient processes.

Based on this evidence, we expected to find the frontal negative ERP activity associated with the prestimulus SME in both stay and switch conditions. Moreover, we expected to observe a different timing of the occurrence of the SME in the two conditions related to the nature of the underlying attentional mechanisms.

In fact, according to the dual network model of attentional control (Dosenbach et al. 2008) sustained and transient attentional processes act in parallel but relatively independent in time. Transient processes are primarily involved in task switching ensuring goal-directed adjustments to the task requirements and sustained processes provide a stable background over the whole epoch. Hence, in stay trials sustained processes reflecting set maintenance would prevail at the beginning of the trial, extending their influence across the entire epoch. In switch trials transient processes would prevail before stimulus onset reflecting an efficient adjustment to the task demands.

## Methods

### Participants

Twenty-one right-handed healthy students (mean age 22.3; four men) participated for course credits. All participants were native German speakers. All had normal or corrected-to-normal vision. The experimental data were collected after obtaining informed written consent from each subject. The study was approved by the local ethics committee. All data were recorded at the Institute of Psychology of the University of Bern.

### Stimulus and materials

Four hundred and thirty-two concrete nouns were selected from a database of written German words (Baayen et al. 1995). The words were composed of 4–10 letters and had a frequency ranging between 1 and 30 occurrences per million. We used nouns that could be judged either as neutral or emotional for an emotional semantic decision task or as animate or inanimate for the non–emotional semantic decision task. The 432 words were divided in four categories of equal size, namely (1) emotional-animate (e.g., aggressor), (2) neutral-animate (e.g., grain), (3) emotional-inanimate (e.g., poetry), and (4) neutral-inanimate (e.g., fork). At study, 2/3 of these words were randomly selected – in equal proportion – from the four categories. At test, the remaining 1/3 were inserted as new words for the recognition test. Sixteen additional words were selected from the same database to create a practice list for the study and test phases. All stimuli were presented in black (font Courier New 24) on a gray background and word length varied between 2.7 and 6.2 cm. The subjects were seated 1.2 m away from the screen and the words subtended a vertical visual angle of 0.4° and a horizontal visual angle ranging between 1.3° and 3.1°.

### Task and procedure

At study, every word was preceded by a cue, which consisted of the presentation of either the letter O or the letter X. After the letter O, the participants had to decide whether the upcoming word was animate or inanimate. Following the letter X, they had to decide whether the upcoming word was neutral or emotional. The cues were randomly presented, ensuring that the task on each trial could not be predicted before the cue. The cues were displayed for 2600 msec. They were followed by a 100 msec blank period and the presentation of the word. Each word was presented for 300 msec, followed by a fixation-cross for 2200 msec. Thus, each trial had a duration of 5200 msec. The subjects were instructed to respond by pressing one of four keys with the index and middle fingers. The middle and index fingers were used, respectively, to respond to emotional and animacy judgments. After 18-min rest, there was a surprise recognition memory test, in which all 288 words randomly presented in the study phase (old words) were used along with 144 new words, divided also into the four above mentioned categories. Before the presentation of each word, an exclamation mark was shown for 1000 msec, serving as a fixation point and as a warning stimulus. The words were visually presented one at a time for 300 msec, followed by a blank screen of 2900 msec. Thus, each trial lasted 4200 msec. Participants were instructed to decide for each word whether they had seen it in the previous experiment, and to indicate whether they were confident or not about their decision. As before, the subjects were instructed to respond by pressing one of four keys with the index and middle fingers. The middle and index fingers were used, respectively, to respond to sure and unsure old/new judgments. In both experiments, speed and accuracy of responses were emphasized, the word sequence was randomized and finger assignment for the responses was counterbalanced.

### EEG acquisition and preprocessing

The EEG was recorded in an electrically shielded and air-conditioned room with Ag/AgCl electrodes mounted on a MR 64 channel electro cap (FMS, Munich, Germany). During the EEG acquisition, the Fz electrode was used as reference and the EEG was sampled at 500 Hz/channel, digitally band pass filtered between 0.01 and 250 Hz and stored with a 500 Hz sampling rate. Offline, we preprocessed the data with Analyzer software (Brain Products GmbH, Munich, Germany), digitally band pass filtered between 0.01 and 16 Hz, corrected for horizontal and vertical eye movements using an independent component analysis. No baseline correction was applied.

For the complete description of the stimuli and materials, task and procedure, EEG acquisition and preprocessing see (Padovani et al. 2011).

### Analysis of behavioral data

In order to analyze the data on the behavioral and neural levels, trials were collapsed across both tasks (emotional and semantic) induced by the cues and separated according to whether the preceding trial contained a word with the same or a different cue instruction.

Mean accuracy and reaction times (RTs) were computed for both experiments. The differences within and between stay and switch conditions were analyzed with two-tailed *t* tests, and the alpha level was set at 0.05. At study, these measures were also related to the later memory performance. To analyze the recognition memory performance in the test phase we used the *Pr* discrimination index (*P*_hit_−*P*_false_ alarm) based on the two high-threshold model (Snodgrass and Corwin 1988) as in our prior study (Padovani et al. 2011).

### ERP analyses

ERP waveforms from each electrode site were averaged across each condition (stay vs. switch) separately for subsequently remembered or forgotten study words. Trials with no response or a response faster than 200 msec were excluded, following the literature (Otten and Rugg 2001; Otten et al. 2006). Furthermore, ERPs were based on a minimum of 12 artifact-free trials. This threshold was based on previous studies focused on encoding-related brain activity (Otten et al. 2006; Gruber and Otten 2010; Padovani et al. 2011). For the calculation of the prestimulus SME, four individual grand-average ERPs were computed for each condition (stay vs. switch) and recognition mode (remembered vs. forgotten). To gain more artifact-free trials and maximize our effect, we decided to exclude the initial 700 msec from the epoch. In fact, according to the literature the prestimulus SME appeared always in closer time correspondence with the target presentation (Otten et al. 2006, 2010; Guderian et al. 2009). Therefore, the analysis window started 2 sec before word presentation and ended at the onset of the word. Encoding-related activity was analyzed dividing this time window in two parts of 1 sec each, since we expected to observe an effect that was lasting less than the entire epoch duration.

In addition, we computed an analysis of variance (ANOVA) for repeated measures in both time intervals on the average amplitudes, on eight frontal electrode sites (Fpz, AF1, AF2, Fz, F1, F2, F3, F4) for each item type. The ANOVA included factors of subsequent memory performance (remembered and forgotten) and electrode sites. These electrodes were selected according to a priori expectations about a frontal distribution of the SME, as reported in the literature (cf. Otten et al. 2006, 2010). To assess the presence of an interaction between performance, condition (switch and stay) and time window (from −2 to −1 sec and from −1 to 0 sec) on the mean activity across the eight frontal electrodes, we have computed another ANOVA for repeated measures with these three factors.

Further analyses explored the SME in the stay condition and contrasted it with the switch condition and were based on methods that assess the significance of an ERP effect across the entire scalp. More precisely, we computed the amplitude differences in each condition and time window with the global field power (GFP) analyses that is a parametric assessment of map strength, computed as standard deviation of the momentary potential values and independent of topography (Lehmann and Skrandies 1980). The resulting amplitude differences indicate a different global strength in similar source distributions. In order to investigate the spatial distribution of the effects, we used TANOVAs (topographic analyses of variance) applied to ERP data averaged across intervals and based on amplitude normalized maps. This was done to obtain a clear distinction between topographic effects and amplitude differences (e.g., Michel et al. 2009). A repeated measures TANOVA was performed in each condition and time window to analyze subsequent memory performance across the 64 electrodes sites. Based on randomization techniques, TANOVA is a powerful nonparametric test for the analysis of multichannel ERP data used to assess global dissimilarities between electric fields. This type of analysis corresponds to an ANOVA with all channels as repeated measures, but has the advantage that it considers all channels as a single entity avoiding a preselection of electrodes, and does not require a correction for multiple testing across electrodes.

Additionally, we have computed a post hoc TANOVA to assess the possible influence on the prestimulus SME of a third factor, instruction type (emotional and semantic) with the two factors already considered in the analyses namely conditions and performance. This factor was not considered in the main analyses for the lack of sufficient trials.

## Results

### Behavioral results

At study, mean RTs were 1025 msec (SD = 157) for stay trials and 1078 msec (SD = 193) for switch trials. In line with the literature, RTs in hit trials were significantly shorter for stay than for switch trials (*t*(20) = −3.12, *P* < 0.005), whereas RTs in miss trials did not differ between the two conditions (*t*(20) = −1.83, *P* = 0.082).

**Figure 1:**
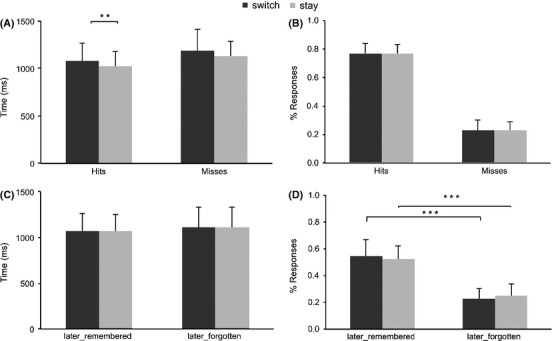
Behavioral measures at study. *T*-test differences: **: *P* < 0.01 and ***: *P* < 0.001. (A) Reaction times (RTs) averaged across subjects. (B) Proportions of responses averaged across subjects. (C) RTs averaged across subjects, related to later subsequent memory performance. (D) Proportion of responses averaged across subjects, related to later subsequent memory performance.

The proportion of correct responses was 77% (SD = 6%) for stay trials and 77% (SD = 7%) for switch trials, showing no statistical difference (see Fig. 1). Additional analyses were computed to evaluate whether the accuracy and time to respond to an item at study were related to later memory performance. In both the stay and switch conditions, responses were more accurate for subsequently remembered than for forgotten words (*t*(20) = 7.40, *P* < 0.001 and *t*(20) = 7.34, *P* < 0.001 for stay and switch trials, respectively) but RTs were not different between conditions (*t*(20) = −1.58, *P* = 0.129 and *t*(20) = −1.68, *P* = 0.109 for stay and switch trials, respectively) (see Fig. 1). The apparent difference between conditions of later remembered items did not reach significance (*t*(20) = −0.97, *P* = 0.342) and no RT differences were found.

**Figure 2:**
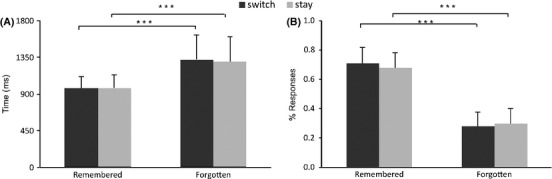
Behavioral measures at test. *T*-test differences: ***: *P* < 0.001. Only confident hits were considered remembered items, whereas forgotten values include nonconfident hits and wrong answers. (A) Reaction times (RTs) averaged across subjects. (B) Proportion of responses averaged across subjects.

At test, the proportion of remembered responses was 68% in the stay condition and 71% in the switch condition, and did not differ between conditions (*t*(20) = −0.93, *P* = 0.364) as well as mean RTs for correct answers (*t*(20) = 0.29, *P* = 0.799). Recognition memory performance results at test are shown in Table 1 and Figure 2.

**Table 1:** Recognition memory performance

	Recognition judgment			
	
Word type	Sure old	Unsure old	Sure new	Unsure new
Proportion of responses				
Old				
Same	0.68 (0.10)	0.05 (0.07)	0.21 (0.11)	0.04 (0.06)
Switch	0.71 (0.11)	0.05 (0.10)	0.19 (0.10)	0.04 (0.07)
New	0.21 (011)	0.05 (0.05)	0.61 (0.16)	0.11 (0.15)
Mean reaction time (msec)				
Old				
Same	974.45 (157)	1501.49 (390)	1157.40 (226)	1521.89 (361)
Switch	972.44 (140)	1639.29 (369)	1138.07 (164)	1530.97 (354)
New	1102.70 (230)	1722.93 (296)	1102.54 (161)	1575.24 (365)

Values are across-subject means (SD). *n* = 21.

Accuracy of confident and not confident recognition was assessed by the discrimination index *Pr* (*P*_hit_−*P*_false_ alarm). For confident hits, the discrimination index *Pr* was 0.43 in the stay condition and 0.49 in the switch condition, which was different from zero (stay condition: *t*(20) = 20.60, switch condition: *t*(20) = 21.66, both *P*s < 0.001). There was no difference between the two discrimination indices (*t*(20) = −1.59, *P* = 0.128).

For nonconfident hits, the discrimination index was not different from zero in both conditions (stay condition: *t*(20) = 0.13, switch condition: *t*(20) = −0.49, both *P*s > 0.620). On the basis of these findings, only confident hits were considered as “remembered” items in the ERP analyses, as they were the only ones that reliably discriminated between old and new words. The reason for this procedure was to maximize the signal-to-noise ratio for SMEs by comparing the ERPs of items yielding confident hits versus those yielding non confident hits or misses (Padovani et al. 2011). The differences in mean RTs and proportion of responses between subsequently remembered and subsequently forgotten items were always significant in the stay (RTs: *t*(20) = −5.05, *P* < 0.001; proportion of responses: *t*(20) = 14.01, *P* < 0.001) and switch conditions (RTs: *t*(20) = −2.34, *P* < 0.030; proportion of responses: *t*(20) = 13.93, *P* < 0.001) (see Fig. 2). There was no difference between the two conditions.

### EEG data

**Figure 3:**
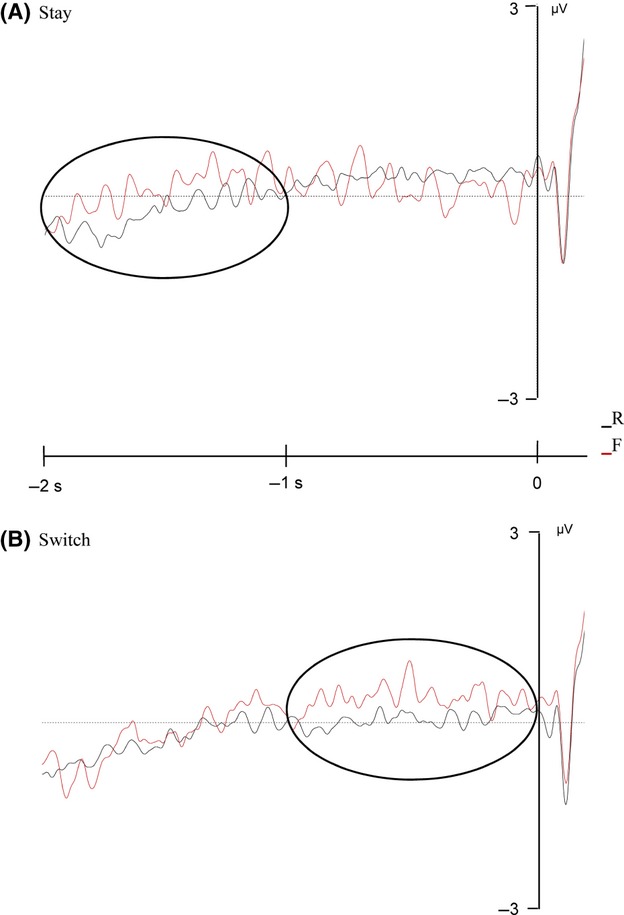
Prestimulus neural activity. R stands for remembered and F stands for forgotten words. Group-averaged event-related potential (ERP) waveforms elicited by prestimulus cues at the representative frontal electrode site Fpz are depicted. Positive values are plotted upwards. The circles represent the time periods used for waveform quantification. The ERPs of subsequently remembered and forgotten words differed in both conditions before word onset according to later memory performance, at different times. (A) Prestimulus activity predictive of encoding success in the stay condition in the time interval from −2 to −1 sec. (B) Prestimulus activity predictive of encoding success in the switch condition in the time interval from −1 to −0 sec.

**Figure 4:**
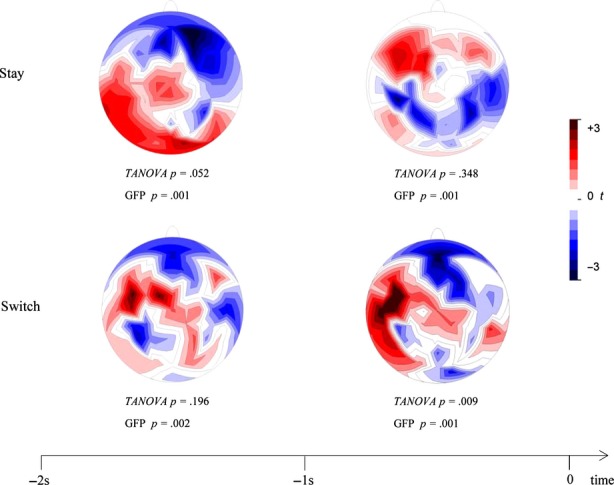
Average *t*-maps of prestimulus SMEs for both conditions and time intervals, showing the distribution of the ERP differences across the scalp. The upper *t*-maps refer to the stay condition and the lower maps to the switch condition. SMEs, subsequent memory effect; ERP, event-related potential.

In the switch and in the stay conditions the items that were subsequently remembered versus forgotten showed a pattern similar to previous studies before the words' onset (Otten et al. 2006, 2010; Padovani et al. 2011). The potentials at frontal electrodes preceding the words that were later remembered were frontally more negative-going than those preceding words that were later forgotten (see Figs. 3, 4). Furthermore, we computed an ANOVA for repeated measures on the average potentials at eight frontal electrodes (Fpz, AF1, AF2, Fz, F1, F2, F3, F4) and compared remembered and forgotten words, for each condition and time window. In the time window between −2 and −1 sec, the analyses yielded in the stay condition a significant main effect of performance, that is, remembered more negative than forgotten (*F*(1, 20) = 5.81, *P* = 0.018). By contrast, in the switch condition this comparison was not significant (*F*(1, 20) = 0.46, *P* = 0.506). In the following time window from −1 to 0 sec, this comparison yielded an opposite pattern: a significant main effect of performance in the switch condition (*F*(1, 20) = 5.22, *P* = 0.033) and no effect in the stay condition (*F*(1, 20) = 1.17, *P* = 0.293). A further ANOVA for repeated measures showed an interaction between time window and performance for the mean potentials at the eight frontal electrodes in the switch (*F*(1, 20) = 4.86, *P* = 0.039) and stay (*F*(1, 20) = 9.87, *P* = 0.005) conditions. We therefore found the previously reported scalp location and direction of the prestimulus SME in the switch and in the stay conditions, nevertheless these varied with the time window.

An additional ANOVA for repeated measures computed on the average activity over the eight frontal electrodes revealed an interaction (*F*(1, 20) = 11.56, *P* = 0.003) between performance (remembered and forgotten), condition (switch and stay), and time window (from −2 to −1 sec and from −1 to 0 sec). Furthermore, this analysis showed a main effect for the factor time window (*F*(1, 20) = 11.20, *P* = 0.003) and a marginal main effect of performance (*F*(1, 20) = 3.75, *P* = 0.067) and no effect of condition (*F*(1, 20) = 2.15, *P* = 0.158). These results indicate a reliable difference over the frontal electrodes sites between the SMEs in the two conditions across the different time windows.

Using global statistics on the scalp electric fields, we measured the performance difference (remembered–forgotten), that is, SMEs, computing the average mean activity in the time window from −2 to −1 sec. Paired TANOVAs for each condition yielded a marginal effect in the stay condition (*P* = 0.052) and no effect in the switch condition (*P* = 0.196). The same procedure was applied in the time interval from −1 to −0 sec and here again, we found an opposite pattern, this means a significant effect in the switch condition (*P* = 0.009) but no significant effect in the stay condition (*P* = 0.348). The spatial distribution of these effects was further displayed and explored on the scalp level with *t*-maps as shown in Figure 4.

Hence, these results suggest that the processing of subsequently remembered and forgotten words might differ in location and/or relative contribution of the brain structures across the entire epoch with an opposite pattern in the two time windows, showing the emergence of the SME in both conditions but in different time frames.

**Figure 5:**
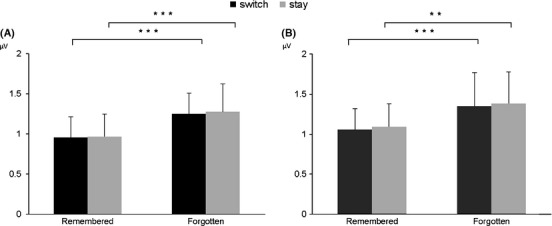
*T*-test differences in global field power (GFP): **: *P* < 0.01 and ***: *P* < 0.001. Note that the standard deviations of the mean values shown do not correspond to the standard deviation employed for the paired *t*-tests. (A) Time interval from −2 to −1 sec. (B) Time interval from −1 to 0 sec.

Differences in amplitude independent of topography were analyzed based on the differences in GFP (see Figs. 4, 5). In the −2 to −1 sec window, we observed that forgotten words were associated with a higher GFP than remembered words both in the stay condition (*t*(20) = −4.47, *P* < 0.001) and in the switch condition (*t*(20) = −4.88, *P* < 0.001). In the interval between −1 and −0 sec, GFP results were similar, showing an effect in both conditions (*t*(20) = −3.54, *P* = 0.002) and (*t*(20) = −4.21, *P* < 0.001) in the stay and switch conditions, respectively. The significant *t* values were in all cases negative, indicating higher prestimulus activity for the subsequently forgotten versus the subsequently remembered items as previously shown (Padovani et al. 2011).

A post hoc TANOVA was computed to assess the possible interactions of a third factor, the instruction type (emotional, semantic) with the two factors already considered in the previous analyses, namely condition and performance. To compute these analyses, we have considered the data of only 14 subjects with a minimum number of 10 trials for each condition. The results showed neither triple interaction nor other effects, but only a main effect near to significance (*P* = 0.06) for condition and performance in the time window from −1 to 0 sec. This finding, taken with caution, provides an indication that collapsing the trial activity across instruction types was correct and confirms the validity of our analyses, although it suffers from a loss of sufficient trials.

## Discussion

The aim of the present study was to investigate which kind of attentional processes contribute to the prestimulus SME and to clarify the nature and the time of occurrence of the preparatory processes that can modulate memory formation. Therefore, we contrasted patterns of electrical brain activity preceding the presentation of words that were later remembered or forgotten in two distinct encoding conditions, using a random task cueing setting, that is, stay and switch trials. These two conditions were characterized either by a repeated task across two or more consecutive trials in the stay condition or by a task switch in the switch condition.

The results revealed a distinct electrophysiological activity for subsequently remembered versus forgotten items (SME) across the entire epoch. More specifically, with local and global types of analyses, we observed different SMEs, namely in the stay condition, during the 1-second window following the cue presentation and in the switch condition, during the 1-second window before stimulus onset. The observed pattern of activity resembled previously reported SME topographies (Otten et al. 2006, 2010; Padovani et al. 2011), suggesting that both sustained and transient attentional processes play a role in the determination of the prestimulus SME occurring in different time periods during task preparation.

Interestingly, these findings highlight the temporal resolution of the activation of the executive networks proposed in the dual network model of attentional control, which can be considered a good theoretical framework to account for these results (Fair et al. 2007; Dosenbach et al. 2008, 2007; Petersen and Posner 2012). These networks support and flexibly regulate top–down control, setting up the basis of the learning process. The model presupposes two parallel control mechanisms with different functional properties mediated by discrete anatomical substrates. The first is represented by the fronto-parietal system and accounts for transient adaptive control in cued delayed target paradigms, as the present one, and is involved in task switching. The second is represented by the cingulo-opercular system that mediates sustained set maintenance and provides an enduring background for task execution across trials. These separate networks are active in rapid and slower timescales supporting adaptability (fronto-parietal system) and stability (cingulo-opercular system) of top–down control (Fair et al. 2007; Dosenbach et al. 2008, 2007). The possibility to sustain task information over time allows maintaining relevant information in order to control and adjust goal-directed behavior according to the task demands (Miller 2000). Consistently, our results show the occurrence of the effect in the stay condition, in the time frame following the cue presentation. This effect appearing at the beginning of the trial can be related to set maintenance that ensures the stability and availability of task sets across the entire epoch. Conversely, the effect in the switch condition might represent the control initiation allowing flexible processing of the relevant information. This effect seems related to a rapid and efficient adjustment to the ongoing task requirements and therefore needs more time to develop and takes place right before the stimulus onset.

According to this model, we found the typical SME topography in the stay condition reaching its peak shortly after the presentation of the repeated cue (in the time window from −2 to −1 sec). In addition, we also expected that this topography would extend across the entire epoch, that is, in both time windows. However, this was not the case. Presumably, the influence of sustained processes on the prestimulus SME in the window preceding the stimulus onset (from −1 to 0 sec) is present but too subtle to be detected, because attenuated by the predominant ongoing parallel activation of the transient activity related to the switch trials, reaching its peak in this time window. In line with this interpretation, the topographic analyses yielded on a global level a stronger effect in the switch condition compared to the effect found in the stay condition; this result is in line with the knowledge that transient reconfiguration processes related to task switching recruit more attentional resources than do sustained attentional processes. The engagement of an higher amount of attentional resources reflects increased demand for cognitive control (Braver et al. 2003), which on a performance level, translates into a need for more time and effort for task execution (Meiran et al. 2000; Corbetta and Shulman 2002; Monsell 2003). Coherently, we find at study longer RTs for switch versus stay trials, revealing a behavioral cost due to additional computations required for task switching. At test, we observed that slightly more words were recognized in switch than in stay trials, although there was no statistical difference between the two conditions.

As previously suggested by Reynolds et al. (2004), such increased demands might be required not only for task switching, but also for maintenance of both task representations in accessible states across trials, together with the additional need to favor and consequently react to the appropriate one. In fact in the same study, the additional attentional load provoked by a task switch setting, similar to the one used in our study, showed poststimulus effects both at a behavioral and neural level, resembling our findings. At study, the behavioral performance was characterized by slower RTs and lower accuracy in the task switching condition. At test, they found that fewer words were recognized in the task switching condition than in the stay condition. On a neural level, Reynolds et al. (2004) showed a higher activation in the prefrontal cortex for switch versus stay conditions.

Interestingly, in a previous study based on the same data set, Braver et al. (2003) reported a double dissociation between transient and sustained effects of attentional control in the activity of several brain areas, during task switching. Transient effects were evident in left PFC and parietal cortex consistently with recent theories of attentional control (Fair et al. 2007; Dosenbach et al. 2008, 2007; Petersen and Posner 2012). Sustained effects were instead shown in right anterior prefrontal cortex and other right lateralized brain regions. This is in line with the right lateralization of the frontal negativity that we found in the stay condition. The temporal dissociability of these processes together with their different neural substrates led the authors to the conclusion that both transient and sustained components of cognitive control are involved in task switching paradigms.

To test if this dissociation would affect memory encoding, Reynolds et al. (2004) investigated also the impact of higher activation levels in the prefrontal cortex on sub-sequent memory and found a positive correlation between higher LIPC activation and subsequently remembered versus forgotten items. The higher recognition rate of stay trials led the authors to propose that, as task switching is more effortful and elicits greater LIPC response, this higher demand reflects encoding under divided attention, in which attentional resources are employed in different processes, namely task goals updating and semantic classification. The interference between these two tasks had a negative impact on memory formation determining a worse retrieval performance under the switch condition.

However, several studies (Brewer et al. 1998; Gabrieli et al. 1998; Wagner et al. 1998; Baker et al. 2001; Otten et al. 2001; Rugg et al. 2002) also showed a correlation between higher event-related responses in prefrontal cortex at encoding and subsequently remembered versus forgotten items. To account for these results, an alternative hypothesis was raised, which states that an increase of processing resources during task switching, together with the additional context related to the semantic nature of the tasks leads to a more enriched memory representation (cf. Otten et al. 2006). The availability of a large amount of processing resources at encoding facilitates task preparation, increasing item distinctiveness and consequently attenuating competition and interference during retrieval (cf. Reynolds et al. 2004). Our results, focused on the prestimulus period are consistent with both interpretations because we do not find a reliable difference at retrieval between switch and stay trials.

A similar design to ours was used by Otten et al. (2010), who observed a prestimulus SME in the switch and stay conditions, switching between visual and auditory modality. Our results confirm their main finding, that is, the involvement of both attentional processes in the generation of the prestimulus SME. However, using different analysis strategies and a different type of paradigm, we could further specify the temporal occurrence of sustained and transient mechanisms, highlighting in the two encoding conditions an opposite pattern of effects, even on a global level.

A notable characteristic of our findings was the prolonged duration of each SME in the two conditions, compared with the SMEs previously reported in the literature (Otten et al. 2006, 2010; Gruber and Otten 2010; Padovani et al. 2011). It indicates that different types of attentional processes contributing to the effect are consistently but selectively active across the trial duration.

The frontal negativity of the switch and stay SME patterns shows a high overlap with previously reported SMEs (Otten et al. 2006, 2010; Padovani et al. 2011). The frontal location of the effects is in accordance with the crucial role of PFC typically found in subsequent memory literature (Polyn and Kahana 2008). Moreover, this pattern is consistent with findings that show the involvement of frontal brain areas in cognitive control processes and more specifically in the establishment of task sets. This is coherent with the hypothesis that the prefrontal cortex is the source of the preconfiguration of appropriate cognitive processes (Sakai and Passingham 2003, 2006; Haynes et al. 2007; Rowe et al. 2007). Similar patterns of activity in PFC have been also shown to be engaged in the formation of a context (Braver et al. 2001; Polyn and Kahana 2008), ensuring a correct reaction to incoming information. In line with these findings, it has been proposed that the sustained and transient attentional mechanisms that maintain and adapt this PFC activity to the task demands might influence PFC in a way that it becomes “the neural seat of temporal context” (Polyn and Kahana 2008).

In conclusion, this study expands our knowledge on the prestimulus SME, specifying the nature and the time course of the attentional processes that interplay with memory formation. The results confirm the crucial role of sustained and transient attentional mechanisms, in distinct consecutive time periods, in the establishment of a “neural context” (cf. Otten et al. 2006). This context is influenced by the temporal resolution of these attentional processes and provides a neural background that enables preparatory processes and modulates positive and negative neural predictors of memory encoding.
